# Reliable Geographical Forwarding in Cognitive Radio Sensor Networks Using Virtual Clusters

**DOI:** 10.3390/s140508996

**Published:** 2014-05-21

**Authors:** Suleiman Zubair, Norsheila Fisal

**Affiliations:** UTM-MIMOS Centre of Excellence in Telecommunication Technology, Faculty of Electrical Engineering, Universiti Teknologi Malaysia, 81310 UTM Johor Bahru, Malaysia; E-Mail: sheila@fke.utm.my

**Keywords:** cognitive radio *ad hoc* wireless networks, cognitive radio sensor network, geographical forwarding, reliable routing, routing protocol, opportunistic routing, virtual clustering

## Abstract

The need for implementing reliable data transfer in resource-constrained cognitive radio *ad hoc* networks is still an open issue in the research community. Although geographical forwarding schemes are characterized by their low overhead and efficiency in reliable data transfer in traditional wireless sensor network, this potential is still yet to be utilized for viable routing options in resource-constrained cognitive radio *ad hoc* networks in the presence of lossy links. In this paper, a novel geographical forwarding technique that does not restrict the choice of the next hop to the nodes in the selected route is presented. This is achieved by the creation of virtual clusters based on spectrum correlation from which the next hop choice is made based on link quality. The design maximizes the use of idle listening and receiver contention prioritization for energy efficiency, the avoidance of routing hot spots and stability. The validation result, which closely follows the simulation result, shows that the developed scheme can make more advancement to the sink as against the usual decisions of relevant *ad hoc* on-demand distance vector route select operations, while ensuring channel quality. Further simulation results have shown the enhanced reliability, lower latency and energy efficiency of the presented scheme.

## 1. Introduction

The introduction of cognitive radios (CR) onto wireless sensor network (WSN) nodes promises not only to improve the utilization of unused spectra, but to solve the crowded spectra issue. It also carries along with it various benefits, which include: the enhancement of channel reliability used under bursty traffic, lifetime maximization via adaptive power and bandwidth allocation and making possible the easy integration of WSN into the heterogeneous Internet of things (IoT) [1,2]. As such, the cognitive radio sensor network (CRSN) paradigm is gaining more attention in the research community [3]. Implementing effective data transmission schemes across the CRSN is core to harvesting these benefits of the CRSN paradigm [4–6]. Although research in routing for CR *ad hoc* networks has been active in recent times, the issue of providing quality of service in terms of reliability still remains a crucial open issue that needs to be addressed [7]. This issue becomes more challenging when the unique resource constraint characteristics of CRSN are to be considered [8].

For CR *ad hoc* networks (CRAHNs), the dynamic nature of channel availability between the source and sink node has made on-demand routing approaches favored over other approaches [9]. Among reviewed on-demand routing protocols for CRAHNs, routing protocols that modify the *ad hoc* on-demand distance vector (AODV) are the most popular ones [10]. However, although the AODV scheme provides an easy way to manage the dynamic nature of the network topology by securing a path to the sink before commencement of the data-routing stage, the common control channel (CC) concept, which is mostly utilized in AODV-based CRAHNs, does not provide a framework for confirming node-to-node instantaneous link quality at the point of packet transmission between nodes [7]. This is because the next hop decision is usually concluded via CC based on the joint route and spectrum selection scheme implemented for most CRAHNs [6]. This can lead to serious adverse quality of service (QoS) effects, especially for resource-constrained geographical forwarding schemes [11].

Geographic routing protocols are classified as efficient in wireless networks specifically for two reasons: (i) transmitting nodes only need to know the location information of their direct neighbors in order to forward packets, and hence, the state stored is minimum; (ii) discovery floods and state propagation are not required beyond a single hop. Thus, they conserve energy and bandwidth. In the greedy forwarding mechanism, which is the main component of geographic routing, each node forwards a packet to the neighbor that is closest to the destination. This strategy can only be classified as an efficient, low-overhead method of data delivery based on three assumptions, namely: (i) accurate localization; (ii) sufficient network density; and (iii) high link reliability independent of distance within the physical radio range. However, while the first two assumptions may be acceptable in some cases, the assumption concerning highly reliable links is unlikely to be valid in any realistic deployment. This is because of the existence of a large region of transition where the link quality has high variance, including both good and highly unreliable links [11]. Consequently, experimental studies on wireless *ad hoc* and sensor networks [12,13], have shown that wireless links can be highly unreliable and that this must be explicitly taken into account when considering higher-layer protocols.

While considering the above, in searching for the next hop in CRSN geographical forwarding based on the joint route and spectrum selection scheme, the choice between two criteria usually arise: (i) the stipulation of the closest node to the transmitting node criterion; or (ii) the stipulation of the closest node to the sink criterion. Although the choice of the first criteria has the capability of assuring node-to-node quality links, it cannot be classified as an efficient solution for resource-constrained CRSN, because, this means a greater number of hops will be required to transmit a packet to the sink. The implications of this choice include amplified end-to-end delays and additional energy incurred for the multiple hop-to-hop communication to the sink. On the other hand, if the closest node to the sink criterion is made, which is the typical greedy forwarding scenario, the existence of unreliable links, which is referred to as the weakest link problem, is encountered. For this strategy, at each hop, the neighbors that are closest to the destination (also likely to be farthest from the forwarding node) may have poor links with the current node. These “weak links“ will usually result in a high rate of packet drops, resulting in drastic reduction of the delivery rate or increased energy wastage if re-transmissions are employed. Thus, the question arises: what is an efficient and reliable way of implementing geographical forwarding for CRSNs?

While reliability via instantaneous channel quality assessment can easily be implemented in traditional WSN [14–16], the contrary is usually the case for CRSN. This is because instantaneous quality can easily be determined in traditional WSN, since both the next hop decision and packet transfer are done on the same channel. Thus, a transmitting node can decide which among its neighbors have the best channel quality at the point of packet transmission. This, in turn, greatly improves the reliability of such a network. Given the rapid dynamic nature of channel quality variation with time, added with primary user (PU) activity, as is the case of CRSN and CR *ad hoc* networks generally, the issue of the instantaneous confirmation of channel quality becomes very challenging for AODV-modeled protocols, because both the channel decision and next hop decision is usually concluded via a separate channel, referred to as the control channel, which is different from the data channel.

As a result of these issues, recent studies have indicated the need for research efforts to incorporate reactive energy-efficient data-transfer components into AODV strategy-based protocols [8]. In line with this need, we have proposed a virtual cluster-based reliable opportunistic routing (ROR) approach to routing in CRSN. Typical to all on-demand routing protocols, where a path is only sought when it is required, and the same is maintained to the end of the transmission process, ROR provides a very robust routing framework that fuses a reactive geographical forwarding scheme into AODV to create a robust scheme that considers the link quality of communicating nodes for data forwarding. Our strategy disconnects between the joint channel and next hop assignment process, which is a prevalent approach in most routing in CR-based *ad hoc* networks [17]. In ROR, all control signaling is done through CC, and the route request phase is used to search for all possible routes from the source node to the sink. The sink selects the best route that offers certain quality of service (QoS) guarantee levels, basically based on bandwidth and delay. At this point, since ROR seeks to integrate a dynamic reactive forwarding process into the AODV anchor-based forwarding scheme, all next hop nodes along the chosen path are regarded as local minimum resolution (LMR) nodes. The route reply process is used to group the surrounding nodes of each LMR node into virtual contention (VC) groups based on the correlation of spectrum opportunities. In the data forwarding phase, a VC group (VCG) receiver-based contention forwarding scheme is implemented. This scheme fundamentally reduces the contention among participating nodes, thereby increasing the chances of successful data transfer across the VCGs to the sink. The scheme is used for each node to decide on forwarding the data packet based on its current state related to the link quality, location and remaining energy level. As a result, the strength of each approach is used to mitigate the weakness of the other. Simulation performance results show how the ROR framework is effectively able to utilize the CR capabilities to improve communication in CRSN when compared with similar solutions.

It is important to note that the contribution of this work is the implementation of an efficient and reliable geographical forwarding for CRSNs. By this, we do not seek to provide another study of the energy and reliability trade-offs pertaining to geographical forwarding, which has been detailed in the literature [18–21], except were necessary. In this regard, this work will be the first that implements lossy link-aware geographic forwarding for CRSNs.

The rest of the paper is organized as follows: In Section 2, we provide a review of related and relevant works on routing in CRSN. Detailed operational functions of the modules that make up ROR are presented in Section 3, along with the system/network model. For verification of the packet forwarding principle of ROR, in Section 4, a theoretical operation analysis is presented along with simulation results. In Section 5, both the performance evaluation of ROR and the comparative evaluation of ROR are discussed. Finally, the paper is concluded in Section 6.

## 2. Related Work

Among the relevant works in this regard is [22], which proposes a cross-layer cognitive routing protocol, which is called the opportunistic service differentiation routing protocol (OSDRP) for dynamic CRNs. The protocol is comprised of the route discovery, route decision, opportunistic routing with transmit power control (ORTPC) and route maintenance modules. The route discovery follows the usual AODV strategy to discover all possible routes from source to sink, after which, the route decision module then chooses the minimum delay route. During the data-transfer phase, the authors sought to switch to a greedy forwarding-based strategy. However, how neighboring nodes become aware of the operating channel of the sending node was never detailed. More importantly, the local minimum case, which is usually a major cause of route failure in such forwarding techniques, was not addressed in the route maintenance module. Rather, the route maintenance was still basically AODV based. Moreover, energy was not considered in the routing metrics. In the case of ROR, although it follows the same method to determine the best route to the sink, it also uses this phase to solve the local minimum issues that can arise during the data forwarding phase by identifying the nodes along the route as local minimum resolution (LMR) nodes, which are finally resorted to in order to forward the data in case the eligible forwarding node happens to be in a local minimum region. In addition, because we are considering CR sensor nodes, energy was a basic routing metric considered.

The most-recent work in this area is the spectrum-aware clustering for efficient multimedia routing in cognitive radio sensor networks (SCEEM) protocol for CRSN [23,24], which was proposed for efficient energy consumption and dynamic spectrum access. SCEEM is based on a hierarchical routing scheme, which organizes neighboring nodes into clusters after the sharing of local spectrum sensing results and residual energy. A cluster head, which usually is the node having the highest energy spectrum rank, is chosen via a self-announcement scheme. The cluster-head is responsible for controlling the spectrum access and data routing. Communication in SCEEM is based on a hybrid time division multiple access (TDMA) and carrier sensing multiple access (CSMA) medium access protocol to relay intra-cluster and inter-cluster packets, respectively. In comparing SCEEM with ROR, both ROR and SCEEM support scalability, because SCEEM is based on hierarchical routing, while ROR is based on geographical routing, and both schemes support scalability [25–27]. Again, both utilize the concept of clustering. However, while SCEEM utilizes clustering for spectrum opportunity aggregation and energy efficiency, ROR utilizes clustering to ensure the instantaneous link quality guarantee and route stability in order to maintain QoS. ROR maximizes the utilization of idle listening, while SCEEM does not take this into consideration. Even though SCEEM utilizes clustering as a means of energy reduction based on the operational principles of radios, SCEEM will expend more energy. This is because in SCEEM, all nodes have to send and receive control messages for clustering and spectrum opportunity aggregation, after which, additional energy is expended in the route request operation. Additionally, it should also be noted that cluster members, as well, take part in forwarding the route request. In contrast, ROR has merged both route request and clustering operations into one operation to achieve QoS. Thus, in ROR, by virtue of position information, nodes can immediately become a cluster member upon hearing the route selection control message. In terms of medium access complexity, SCEEM utilizes a hybrid MAC, which uses TDMA for intra-cluster communication and CSMA for inter-cluster communication. In the intra-cluster communication phase, all nodes broadcast their spectrum opportunities along with their residual energy to their neighbors via CC. Each node then computes its cumulative spectrum energy rank with each of its neighbor and also computes the rank for all of its neighbors. It then compares its rank against all the neighbors' ranks, and if it is found among the three highest ranks, then it becomes a potential cluster-head, after which, all potential cluster-heads compete for heading the cluster by way of announcement. All nodes then synchronize their timers with their cluster-heads, which then assigns transmission slots to each member via the beacon, which also instructs members on which channel to transmit. On the other hand, ROR uses the simple CSMA to achieve an efficient receiver contention prioritization scheme, which significantly reduces nodes' contention, and by effect collision. This is achieved by giving more priority of transmission to the nodes in the closest virtual contention cluster to the sink via the implemented VCG initiative determination and receiver contention prioritization schemes of ROR. The VCG initiative determination procedure is used for each node in eligible VCGs to decide on participating in communication based on its current state related to link quality, location, buffer level and the remaining energy level. This not only simplifies the communication, but also makes the routing distributive by way of avoiding the creation of routing bottle necks, as in the case of SCEEM, which aggregates huge multimedia data at the cluster head and sends the same to the sink via a single route.

Another relevant work in this regard is energy- and cognitive-radio-aware routing (ECR) [28], which is basically a routing protocol designed for CRSNs. SCEEM and ECR are similar in that both adopt the same hierarchical network architectures, which features a cluster head that coordinates the clustering operation, and the route search algorithm to the sink adopts similar principles. However, how the cluster formation was achieved in ECR was never mentioned, which makes implementation impossible. Looking in detail at ECR, during the route request phase, the route request (RREQ) packet is sent as a broadcast towards the sink through a common control channel. Intermediate nodes forward the RREQ based on the channel correlation with the sending node, energy threshold and channel availability criteria. When multiple routes are found, the sink chooses the route with the least number of hops and further assigns the operating channel to individual nodes to reduce channel switching during the data-transfer phase. Route maintenance is only performed locally if the affected node is in close proximity to the sink. Otherwise, a message must be sent back to the source to initiate a new route request, which can be costly. The issues that are left to be solved in this case are, as we have already hinted in the introduction, that the final route decision cannot be justified as the optimal route at any time after the first decision, because of the nature of the CR environment. This case becomes more pronounced when a node in the original route suddenly experiences heavy noise due to the dynamic CR environment, which can lead to increased delays and packet drops. Based on the strategy adopted in ECR, the network is highly prone to experiencing multiple new route requests, which greatly affects the efficiency of the protocol. Moreover, the channel availability metric was not properly accounted for in ECR. On the contrary, the ROR strategy ensures that the best node at each point in time forwards a packet once it belongs to the VC group (VCG) of the eligible nodes.

Although spectrum and energy-aware routing (SER) [29] is not specific to CRSNs, the fact that it considers energy as a routing metric makes it classifiable as a relevant CRSN solution. The protocol presents another modification of the AODV protocol and differs from the others based on its distributed joint routing and channel time-slot allocation strategy for each link. Again, like in other works, carrier sense multiple access with collision avoidance (CSMA/CA) is used at each link for channel access. This protocol excels in energy consumption network balancing and the reduction of contention in the MAC between nodes by its ability of decomposing traffic over different channels or time-slots. However, a detailed implementation method of the MAC component is lacking, which leaves the assumptions open for verification. Although ROR also uses the CSMA technology, CSMA is carefully regulated by introducing a VCG-based initiative determination forwarding scheme that limits contention at the MAC layer and, thus, greatly reduce collision, which is, also, a major concern in wireless sensor-based networks.

Reactive routing for mobile cognitive radio *ad hoc* networks (CAODV) [17] is also a modification of AODV. Although the CAODV is also designed for reactive routing to ensure link quality, the proposed scenario is only suitable for nodes having multiple transceivers. Although the authors claim that the assumptions of the algorithm also hold for single radio scenarios if specific underlying channel coordination mechanisms, like [30], are applied. However, the applicability of this claim is open for proper verification. This is because the layout presented in [30] requires all nodes to negotiate and decide on data channels via a common control channel. Thus, if this is applied to CAODV, the issue of deciding on channels without ascertaining their quality will persist. Hence, CAODV will have to fall back to multiple transceivers to achieve instantaneous quality information at the point of route, which is not a favored design requirement for CRSNs.

Other related works include the probabilistic routing protocol based on prior information (PRP) [31], which expands upon the Dijkstra routing algorithm by introducing a routing metric that enables the nodes to select channels and routes based on the documented performance of the channels during previous transmissions. The metric is formulated based on naive Bayes inference and uses an m-estimate probability to make the route decisions more realistic. The source node first broadcasts a route discovery packet. This packet is disseminated across all the nodes to the sink. This packet enables individual nodes to calculate the cost function of choosing any of its neighbors based on the formulated routing metric. At this stage, channels are tagged to neighbor nodes. During the process of routing, when the PU arrives, the affected node can easily change the path without jeopardizing the entire routing process. Notwithstanding the ability of the network to reconfigure the route, the energy required to implement the Dijkstra routing algorithm can prove to be non-trivial, especially in the case of CRSN. Distributed best-route selection for multipath routing (DBMR) [32] investigates multipath routing selection in performance optimization under energy-constrained CRAHNs via distributed and heuristic routes selected by secondary users (SUs) with the aim to improve end-to-end delay, while taking energy into consideration. Multipath routing is modeled as a restless bandit stochastic process optimization problem that allows secondary users to select routes considering the dynamic occupancy of a licensed spectrum and energy based on a finite-state Markov chain (FSMC) model. This solution also offers protection to the PU transmission by varying the SU's transmission power in each hop with respect to the PU's occupancy in each channel.

## 3. ROR: Reliable Opportunistic Routing for CRSN

In this section, we present the details of ROR based on its major operational blocks after describing basic system assumptions and the network model. ROR consists of the route request phase, route selection phase, VCG formation phase, VCG-based initiative determination forwarding (VIF) and the route management phase, which explains how ROR deals with the local minimum resolution.

### 3.1. System and Network Model

In cognitive radio sensor networks, like in CR networks, generally, primary users have more privileges to spectrum usage than the secondary users; in this case, the CRSN nodes. Thus, the secondary user (SU) nodes dynamically sense the spectrum holes (channels) and switch to a negotiated channel, which is free of primary user (PU) transmission or interference for data transfer.

Nodes are assumed to be location-aware via an on-board GPS or by running a localization algorithm [33]. No specific topology is assumed, and nodes are assumed to be stationary. The nodes are also assumed to have a single half-duplex CR transceiver that can be tuned to any licensed channel. The PU or SU transmit power decays with distance based on the free-space path loss model. The considered model is according to the probabilistic wireless network simulator [34], which considers the signal-to-noise ratio (SNR) for each receiver and transmitter pair and a value of receiver noise variance (RNV). A signal can only be received or detected if the power limit from both neighbors (*i*.*e*., SUs) and primary users (PUs) is above a threshold, *λ_su_* and *λ_pu_*, respectively.

It is assumed that the energy detection sensing mechanism is used in a non-fading environment for PU detection. Thus, when the SU performs spectrum sensing to detect the PU activity, the nature of the received signal, *S*, can be represented as follows [35]:

(1) $$S_{\text{rec}}^{su}=\{\begin{array}{ll} n(t) & \text{if}\;H_{0}.\\ n\left(t\right)+S^{p}(t), & \text{if}\;H_{1}. \end{array}$$

where *H*_0_ represents the PU hypothetical ideal state, while *H*_1_ represents the active state. *n*(*t*) is a zero-mean additive white Gaussian noise (AWGN) and *S^p^*(*t*) represents the PU signal waveform. Accordingly, the probability of the SU detecting the PU, *P_d_* and the probability of a false alarm, *P_f_*, is computed as [35]:

(2) $$P_{d}=\mathrm{Pr}\{Y>\lambda_{pu}|H_{1}\}$$

(3) $$P_{f}=\mathrm{Pr}\{Y>\lambda_{pu}|H_{0}\}$$

where *Y* represents the decision statistic obtained from the energy detection algorithm and λ*_pu_* is the decision threshold. It thus implies that a low value of *P_d_* increases the probability of PU interference, while a high value would result in low spectrum utilization, because false alarms increase the number of missed opportunities.

It is assumed that PUs are available in the network area and that they make use of the licensed spectrum according to a probabilistic model. The PU activity is modeled according to an ON and OFF Poisson state transition model [36,37], in which both states represent the arrival or absence of the primary user, respectively, with *τ_on_* seconds for the ON state and *τ_off_* for the OFF state. These states are exponentially distributed, because the PU arrival is independent [36]. The ON state transits to the OFF state with a probability, *P_on_*, and reverses from the OFF state with a probability, *P_off_*. When the licensed user arrives, it stays for a time period of $T_{PU}^{\text{off}}$.

(4) $$P_{on}=\frac{\tau_{on}}{\tau_{on}+\tau_{\text{off}}}$$

It thus derives that, when the value of *P_on_* is high, there is more PU activity in the licensed spectrum and less opportunity for SU transmission. When *P_on_* is low, which also translates to a high value for *P_off_*, this means the SU has more opportunity to make use of the licensed spectrum. The probability of being in an OFF state is calculated as:

(5) $$P_{\text{off}}=1-P_{on}$$

At the data channel, once the SU has commenced transmission; it completes sending its packet before releasing the channel, and any interference that might arise thereof is controlled by adopting suitable SU transmission power [38].

For simplicity, we assume that a specific frequency band is chosen as the operation area for the sensors with *N* channels having same bandwidth. All control negotiations is carried out via a dedicated common control channel (CC). By default, the transceiver is tuned to CC and only changes during the data-routing phase.

**Figure 1.**
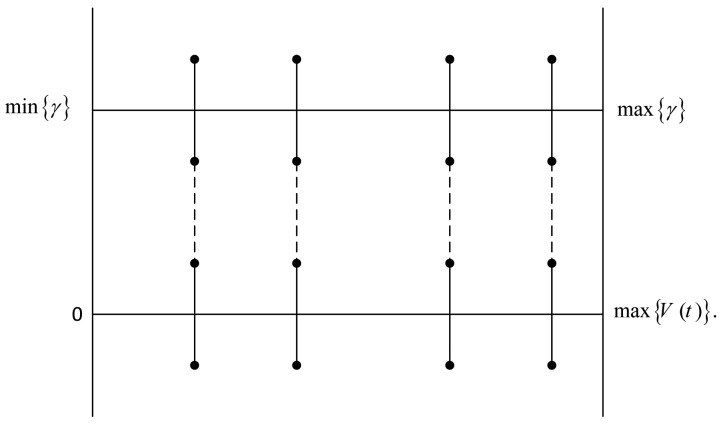
Spectrum quality initiative scaling.

The scheme utilizes a fixed sensing period to sense the potential list of *N* channels. Given the accumulated PU interference level, *γ_i_*, on channel *C_i_*, *i* ∈ *N_c_*, at time instant *t*, the channel quality of $C_{t}^{i}$ is better than that of $C_{t}^{j}$ if *γ_i_*(*t*) < *γ_j_*(*t*), *j* ∈ *N_c_*. This output of the sensing result, $\gamma_{i}^{t}$, is mapped onto an absolute initiative scale, $\upsilon_{i}^{t}$, as shown in Figure 1. Thus, over time, $\upsilon_{i}(t)=\upsilon_{i}^{t_{1}}+\upsilon_{i}^{t_{2}}+\ldots$. In this manner, the quality ratings for each channel, *i* ∈ *N_c_*, are graded according to the scale, *V_i_*_∈_*_N_c__*(*t*). Then, by rearranging the channels in a descending order of *υ_i_*(*t*), the channel with the best quality is easily identified.

Over time, this result is updated on the scale to grade the channel having the greater initiative to be selected for the routing operation depending on the routing requirements. This is because a higher value indicates a low PU activity on that channel and *vice versa*. It is important to note that, once a node has engaged in a successful route setup operation, all sensing operations cease to be performed, and the nodes uses its previous acquired knowledge of the spectrum for data forwarding. Upon this model, ROR is designed to perform routing based on its operational components, which are explained next.

### 3.2. Channel Selection and Route Request Initiation

It is assumed that an event occurs in an event area with an event radius of *r_evt_* meters, and each source node is expected to report its event information to the sink. An event in this case can be motion activated sensors strategically positioned to monitor an area of interest. They are expected to transmit their data, which could be pictures, temperature, humidity or a combination of all to a remote sink.

In order to locate all possible routes from the source to the sink, the source nodes located at the event area compete to initiate the route request operation. Once one successfully sends its route request, all other nodes in the event area refrain from sending and participate in the route request phase. Thus, the source node broadcasts the route request (RREQ) packet, which carries the source address, the sink address and the RREQ identification. The source node then sets a timer, $\bar{T_{r}}$, which is the maximum route negotiation time, within which it expects a route reply packet via the control channel. However, if no reply is received by the source node and it time-outs, then another RREQ is sent. The route request payload carries the important quality of service requirements needed to service the traffic at hand. These include the minimum required data rate required to service the available packets, as stipulated by the application layer. It is pertinent to mention that a high value of *υ_i_*(*t*) does not imperatively mean that the channel can service the application at hand. This is because the *υ_i_*(*t*) does not consider other metrics, like bandwidth and spectrum sensing error, which are very important metrics to be considered, especially for quality of service-specific applications. Thus, it is necessary to confirm the data rate of the chosen channel to be sure it can support the application. The requisite data rate is defined as the achievable data rate, *R*, on any channel at any time instant, *t*. This is the sum of the rate, *R_s_*(*t*), when a channel is detected as idle with the possibility of the appearance of the PU during the packet transmission period, *T_su_*, with the rate achieved, *R_f_*(*t*), as a result of false channel availability. In both cases, the interference among SUs, *I_su_*, has to be considered. Thus, when the PU is inactive, and there is no false alarm of inferring the received signal as a PU transmission. The attainable data rate at a truly detected idle channel is:

(6) $$\begin{array}{ll} b_{s}(t) & =\beta \log_{2}\left(1+\frac{S_{\text{rec}}^{su}}{n(t)+I_{su}}\right)\\ R_{s}(t) & =\left(P_{\text{off}}-P_{f}\right)\frac{T_{su}-\bar{T_{s}}-\bar{T_{r}}}{T_{su}}b_{s}\left(t\right) \end{array}$$

However, a PU can arrive at any time instant during the time, *T_su_*, thus causing interference that eventually converges to (1 − *P_off_*) *T_su_* with the probability $1-e^{-\theta \frac{T_{su}}{\tau_{on}}}$, where *θ* is the scaling factor. On the other hand, the achievable data rate, *R_s_*(*t*), in the situation where PU is active, but it is not detected by the SU, due to spectrum sensing error, is:

(7) $$\begin{array}{ll} b_{f}(t) & =\beta \log_{2}\left(1+\frac{S_{\text{rec}}^{su}}{n(t)+S^{p}(t)+I_{su}}\right)\\ R_{f}(t) & =\left(P_{on}-P_{d}\right)\frac{T_{su}-\bar{T_{s}}-\bar{T_{r}}}{T_{su}}b_{f}\left(t\right) \end{array}$$

Thus, the achievable data rate, *R*, on any channel at any time instant, *t*, is:

(8) $$R(t)=e^{-\theta \frac{T_{su}}{\tau_{on}}}R_{s}\left(t\right)+(1-e^{-\theta \frac{T_{su}}{\tau_{on}}})\;R_{f}\left(t\right)$$

where $e^{-\theta_{1}\frac{T_{su}C}{\tau_{on}}}$ is the probability that the PU remains active on the channel over the entire packet transmission time *T_su_*.

**Figure 2.**
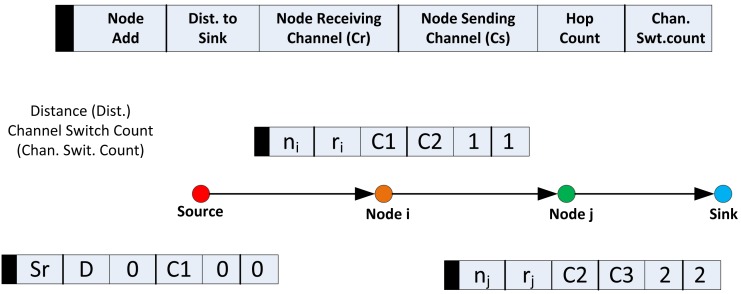
Route request (RREQ) operation with payload.

Hence, the source node places the minimum required data rate needed to service the packets at any instance in the route request payload. Along with this, it also includes its distance to the sink and its data channel of choice in the payload. As illustrated in Figure 2, it is important at this point to note that for the source node, both the node receiving channel, Cr, and node sending channel, Cs, fields will hold the same channel. However, in the case of all nodes after the sink, the Cr and Cs fields may vary, as will be discussed in the following section. The source node then sets both the hop count and the channel switch count to zero and broadcasts the packet through CC.

### 3.3. Route Request

When a node, *i*, receives a RREQ packet from a node, which we will refer to as the requesting node, it checks the packet identification number to be sure it has not beforehand received the same packet beforehand. The node drops the packet if it was previously received. Else, it will only broadcast this RREQ packet if its eligibility (*El*) determination operation returns a one. The eligibility determination is a binary operation that is determined as follows:

(9) $$El=\{\begin{array}{cc} 1, & \{\begin{array}{ll} d_{sk} & <d_{sk}^{\text{req}}\\ E_{th} & \geq E_{th}^{\text{min}}\\ \text{clc} & =clc_{r\text{req}}\\ R(t) & \geq R_{t} \end{array}\\ \text{else}=0 \end{array}$$

For the *El* operation to return a one, four conditions have to be met, which include the following. Node *i* checks to be sure if its own distance to the sink, *d_sk_*, is smaller than that of the requesting node, $d_{sk}^{\text{req}}$. If Node *i* is closer to the sink, it proceeds to check the next condition, else, Node *i* drops the packet. This condition is to ensure that the packet always maintains a forward flow towards the sink. The second condition, $E_{th}\geq E_{th}^{\text{min}}$, ensures that the remaining energy of a node, *E_th_*, is above a minimum value, $E_{th}^{\text{min}}$. The third condition, *clc* = *clc_rreq_*, helps Node *i* check if it has the offered channel available by the requesting node in the *clc_rreq_* field of the RREQ packet. If *clc_rreq_* is available for use from its available list, *clc*, Node *i* proceeds to check the next condition. Else, Node *i* refrains from broadcasting the RREQ packet. The last condition, *R*(*t*) ≥ *R_t_*, is to help Node *i* confirm if the chosen channel in *clc_rreq_* can support the needed traffic at its position. If the channel in the RREQ payload passes the local rate test at Node *i*, Node *i* retains the channels in *clc_rreq_* as the data channel and will not increment the channel switch count. If, however, there exists in its pool another channel with better quality performance than the channel offered by the requesting node, Node *i* replaces the same with its own in the RREQ payload and then increments the channel switch count. In that case, Node *i* will have different channels for both upstream and downstream communication. Node *i* then caches both the channel(s) and the requesting node's position to the sink. The requesting node will later be known as its upstream LMR node if the path is finally selected by the sink as the chosen path. Before Node *i* broadcasts the RREQ packet, it will also replace the requesting node's distance to the sink with its own, increment the hop count and then broadcast the packet. If Node *i*, however, fails any one of these tests, it refrains from broadcasting the RREQ packet. This strategy, among other advantages, keeps the RREQ packet short, unlike in [28], wherein the RREQ packet size progressively increases with the hop count. This route request process continues until all disjoint possible routes to the sink are discovered.

### 3.4. Route Selection

After the sink receives the first route request, it sets a timer. All route requests that arrive at the sink after the timer runs out are classified as out-of-date and are discarded. This is because it can be relatively judged that the late arrival of such packets is an indication of the inferior quality of the corresponding routes; thus, they are immediately dropped. In selecting the final route, two major metrics are considered, namely the hop count and the channel switching count. These metrics are formulated into a route cost function, $R_{i}^{cf}$, that assigns half the weight of the hop metric to the channel switching metric in order to prefer routes that offer less channel switching,

(10) $$R_{i}^{cf}=\omega_{h}\cdot W_{h}+\frac{\omega_{s}}{2}\cdot W_{s}$$

where *ω_h_* and *ω_s_* are the assigned weights for the hop count and the channel switching counts along the route, respectively. While *W_h_* and *W_s_* represent the collected values of hop counts and the channel switching count, respectively. The route with the lowest cost is chosen as the final route, and all nodes along this route are referred to as LMR nodes.

### 3.5. VCG Formation

**Figure 3.**
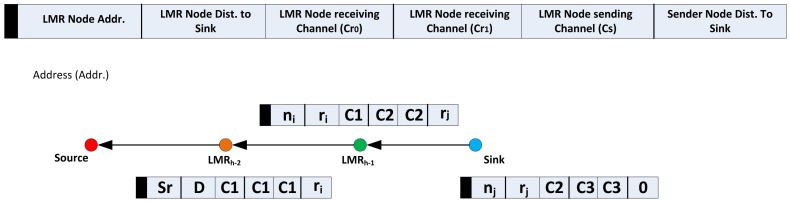
Virtual contention group (VCG) formation operation with payload. LMR, local minimum resolution.

After the route selection, all the nodes along the chosen route are now referred to as local minimum resolution (LMR) nodes. Accordingly, from the sink, the next node in the route is referred to as *LMR*_(_*_h_*_−1)_, *LMR*_(_*_h_*_−2)_, …, *LMR*_0_. The sink then generates and broadcasts the VCG formation packet via CC along the chosen route to the source node. The important fields of the VCG payloads are as shown in Figure 3. All nodes that receive the VCG packet, which is identifiable by reading the VCG packet identifier, are programmed to read and take proper action, and only the LMR node whose address is identified is allowed to forward the broadcast along the upstream to the source node.

Before broadcasting the VCG formation packet, the sink places the next upstream LMR node, (*LMR*_(_*_h_*_−1)_), address, *n_j_*, and its corresponding distance, *r_j_*, from the sink into the VCG formation payload. You will recall that this information has already been acquired during the route request phase. Other required information to be stored in the VCG payload are the sending nodes' distance to the sink; in this case, since the sending node is the sink, the value for this field will be zero. As has been earlier discussed in Section 3.3, there is the possibility that a node might have different channels for upstream and downstream communication. At this point, it is important to note that the Cr0 and Cr1 fields will be respectively replaced with the Cr and Cs information acquired during the route request; while the Cs field will retain the value of Cs, as already acquired from the route request. The significance of this configuration is that it expands the possible reception area, which has the potential of reducing the final hop count. This is because, based on geographical forwarding schemes that make use of both distance and the signal-to-noise reception ratio as forwarding parameters, it has been observed that nodes in the transitional region usually exhibit the highest (joint distance and SNR) forwarding values for lossy links [18], as is the considered case. Finally, the required data rate is also included in the payload, as illustrated in the VCG formation payload in Figure 3. It is important to mention the rationale behind all the identified fields of the VCG payload. Thus, the VCG packet identifier is to differentiate the control packets and help all nodes in the region between consecutive LMR nodes to perform the appropriate VCG formation operation, which shall be discussed shortly. The next upstream LMR node address is to specify the only node with the right to rebroadcast the VCG formation packet. Both the sender node's distance to sink and the LMR node's distance to sink is to enable the other nodes that are not part of the selected route, but exist in the region between two LMR nodes, to determine their position with respect to the sending node and the next upstream LMR node. It is important to note that, although the sink node is not an LMR node, however, with respect to this phase, it can be regarded as an LMR node for simplicity.

Afterwards, all nodes that receive the VCG packet determine its position accordingly. If the nodes' locations fulfill the criteria, $d_{sk}^{lmr}\geq d_{sk}\geq d_{sk}^{\text{snd}}$, then the node proceeds with the next operations of the VCG eligibility operation, as discussed below. Where $d_{sk}^{lmr}$ is the distance of the next upstream LMR node from the sink, $d_{sk}^{\text{snd}}$ is the sending node's distance from the sink and *d_sk_* is the receiving node's distance from the sink.

**Figure 4.**
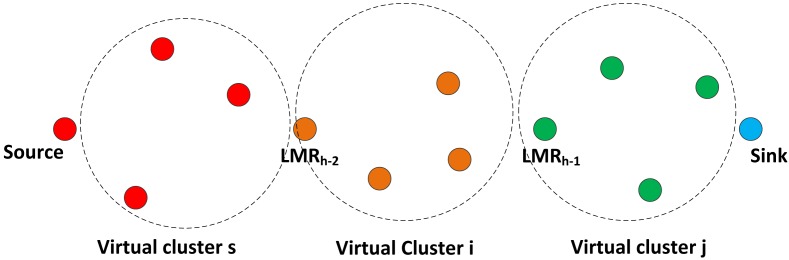
VCG organization after the VCG formation stage. VCG, virtual contention group; PU, primary user.

Any node that receives the VCG formation packet performs a VCG eligibility operation, which is similar to the one done during the route formation phase, as presented in Section 3.2 above; the only difference being that the first condition, $d_{sk}^{lmr}\geq d_{sk}\geq d_{sk}^{\text{snd}}$, which ensures that eligible nodes that will return a one for the VCG-based eligibility operation have to be located in a position between the broadcasting node and the next downstream LMR node. This condition is considered alongside all other three criteria, as in Equation (11). Once a node gets a one for its VCG eligibility operation, the node automatically becomes a member of the virtual cluster of the LMR node's address indicated in the VCG payload, as illustrated in the Figure 4. All eligible nodes then read the corresponding channel assignments and cache them in their routing tables, after which, the node will switch its transceiver to the channel, Cr0, in preparation for the data routing phase.

(11) $$El_{\text{veg}}=\{\begin{array}{cc} 1, & \{\begin{array}{ll} d_{sk}^{lmr} & \geq d_{sk}\geq d_{sk}^{\text{snd}}\\ E_{th} & \geq E_{th}^{\text{min}}\\ \text{clc} & =clc_{r\text{req}}\\ R(t) & \geq R_{t} \end{array}\\ \text{else}=0 \end{array}$$

**Figure 5.**
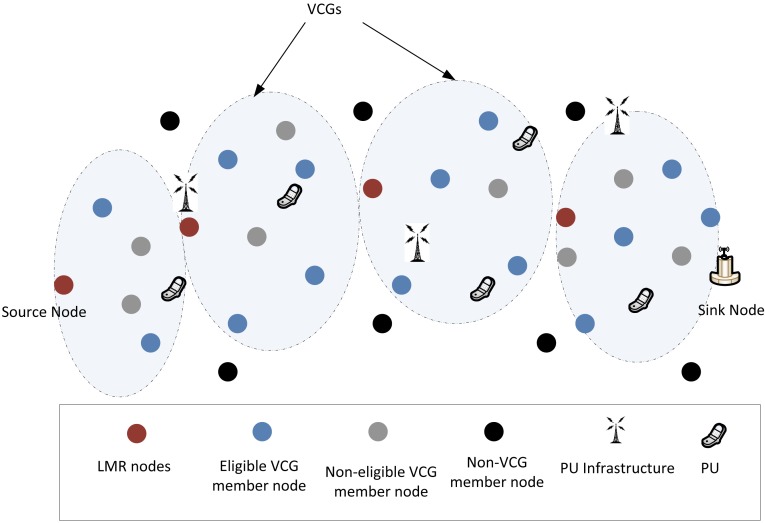
Full network illustration for a reliable opportunistic routing (ROR)-based cognitive radio sensor network (CRSN).

The next LMR node that receives the VCG formation packet inserts the necessary VCG formation information with respect to its location and broadcasts the VCG formation packet. After this operation, it also switches its transceiver to its Cr. In this manner, all the VCGs will be formed, until the VCG formation packet gets to the source node. It should be noted that due to the relatively high density deployment mode characteristic to WSN networks, there will also be a reasonably high correlation of channel quality among VCG proximity nodes [39]. The LMR node periodically geocasts a still-alive packet to its member nodes via its operating channel to indicate that the VCG region is still active. At the end of the initialization phase, the network will look as illustrated in Figure 5.

### 3.6. VCG-Based Data Forwarding Initiative Determination

At this point, ROR switches to its reactive routing component, which is a VCG receiver-based contention forwarding scheme. After switching to its operation channel, as determined from the previous VCG formation operation, a node having the packet to send will broadcast the data packet, which also contains its location information from the sink and the location information of its VCG LMR node from the sink in its header. All eligible VCG nodes that receive the data packet will perform the data initiative forwarding determination (DIFD) operation, which is also a binary operation. A node performs the DIFD operation as follows:

(12) $$I_{\text{DIFD}}=\{\begin{array}{l} 1,\{\begin{array}{ll} d_{sk} & \geq d_{sk}^{fd}\\ B & \geq B_{th}\\ \xi_{\text{pkt}} & \geq \xi_{th}\\ R(t) & \geq R_{t} \end{array}\\ \text{else}=0 \end{array}$$

For the *I_DIFD_* operation to return a one, which signifies the node's initiative, the receiving node's distance with respect to the sink, *d_sk_*, must be lower than the forwarding node's distance to the sink, $d_{sk}^{fd}$. This condition is to ensure that the packet is always routed towards the sink and to prevent packets from infinite routing loops. The second condition, *B* > *B_th_*, which is the local congestion control metric, ensures that the buffer occupancy level of a relay node, *B*, does not exceed a specific threshold, *B_th_*, which is a packet size above the maximum buffer length of a node. This is to ensure that the node does not experience buffer overflow. This is primarily to prevent local congestion and to ensure load distribution among potential eligible relay nodes. This is because there is the possibility of a node becoming a favorite relay node, due to its position in the network. If such a situation occurs, a hot spot is thus created. This means that much of the generated traffic will be favorably routed through this node. Consequently, this can easily result in buffering overflow in such relay nodes. The routing hot-spot scenario can have serious effects on the end-to-end performance of QoS-based applications, like video streaming. Thus, with this condition, *B* > *B_th_*, a node will only accept to relay the packet at hand if its buffer occupancy level, *B*, is above a specific threshold, *B_th_*, thereby giving the chance to other eligible nodes to relay the packet to ensure network load distribution. The third condition, *ξ_pkt_* ≥ *ξ_th_*, is used as a link quality indicator. To ensure a reliable link for the transmission, it is required that any forwarding node must receive the data packet with a signal-to-noise ratio, *ξ_pkt_*, above a threshold, *ξ_th_*. Else, the *I_DIFD_* operation will return a zero, which means the node does not have a reliable link to forward the packet. If, however, the *I_DIFD_* operation returns a one, then the node delays sending the generated acknowledgment (ACK) packet according to a receiver contention prioritization, as explained next.

### 3.7. Receiver Contention Prioritization

The receiver contention prioritization is based upon a priority scheme, which gives more priority to nodes that make more progress towards the sink to forward the packet. Nodes with longer progress have higher priority over other nodes. Based on the location information, the region is divided into Q priority regions, *i*.*e*., *A_i_*, *i* = 1, 2, *Q*. All nodes that pass the *I_DIFD_* operation determine its priority region based upon the location information in the header of the data packet. Next, each node delays sending its ACKpacket, as explained next.

**Figure 6.**
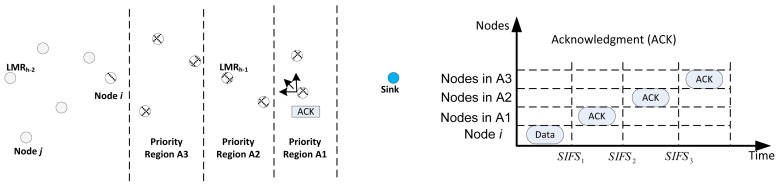
Receiver contention prioritization and backoff scheme.

Each priority region, *A_i_*, corresponds to a delay window size of short inter-frame space (SIFS), *SIFS_i_*. Thus, a node will delay $\Sigma_{j=1}^{i-1}SIFS_{j}+sifs_{i}$ where *sisf_i_* is randomly chosen, such that *sifs_i_* ∈ [0, *EIFS*], where the extended inter-frame space (*EIFS*) = *SIFS_i_* − *SIFS_i_*_−1_,∀*_i_*. Based on this prioritization, only nodes in the same priority region will compete in sending the ACK. Once a node wins and sends the ACK packet, when other nodes that are undergoing the delay overhear this packet, it means an appropriate forwarder has been selected; as a result, they refrain from contesting and drop the packet. This is as illustrated in Figure 6, wherein a case for three priority regions is presented.

Assuming Node *i*, has the data packet to forward, it broadcasts the same on the data channel; all eligible nodes that passed the *I_DIFD_* operation will initialize their SIFS according to their respective regions, as illustrated in Figure 5. According to the scheme, the nodes in the region, *A*_1_, will compete in sending their ACK packet first after the expiration of time *SIFS*_1_. If one node wins, all other nodes refrain from sending the acknowledgment and drop the data packet. However, if no ACK is heard by the nodes at the expiration of the time, *SIFS*_2_, the nodes in *A*_2_ will then compete in sending their ACK packet, and so on. It should be noted that the possibility of two nodes sending an ACK packet without hearing each other cannot arise, because all contending nodes have already been organized in VCGs. The other case that can arise in such a scheme will be the possibility of the collision of ACK packets arising from the same priority region. This can lead to the sending of ACK packets from nodes in lower priority regions, thus leading to the selection of such a node as the next forwarding node. For the fact that the cost of trying to resolve this outweighs the gains, ROR does not seek to resolve this problem. Moreover, the segregation of the nodes into contention regions makes the probability of such an occurrence very low.

However, it is still possible that Node *i* does not receive an ACK, because the eligible node suddenly finds itself in a local minimum area, due to the failure of its neighboring nodes. If the node still receives no response after *k* retries, it determines that a local minimum is reached and switches to its route management module to resolve the issue, as explained next.

### 3.8. Route Management

Node *i* resolves the local minimum problem by checking the distance to the sink of the node from which it previously received the packet (assume Node *j*) with that of its virtual cluster's LMR node. If Node *j* is closer to the sink than its' LMR node, Node *i* sends back the packet to Node *j*, piggybacked with a local minimum alert message. Upon successful reception of the packet by Node *j* or the LMR node, Node *i* changes its status to a non-eligible VCG member node. On the other hand, Node *j* or the LMR node will seek for a new path for the packet by performing the VCG-based data forwarding initiative determination, followed by the receiver contention prioritization, as earlier explained in Section 3.5 and Section 3.6, respectively.

In the event of the failure of an LMR node, which is detected if the VCG members fail to receive the still-alive packet after *z* consecutive times, the VCG node closest in position to the previous LMR node is selected as the next LMR node. To achieve this, each member of the VCG will run a random NLMR timer with a value between *CW*_min_ and $\left(CW_{\min}+{\left(\frac{CW_{\min}}{2}\right)}^{a}\right)$. Upon the expiry of the timer, the node pronounces itself as the new LMR node, reflecting the nodes proximity to the previous LMR node. The closer it is, the faster it pronounces itself. It is important to note that since the LMR nodes do not necessarily take part in packet forwarding, the failure of the LMR node is also a rare occurrence. This makes the scheme a robust one with the ability of sustaining a routing path for as long as is feasible.

### 3.9. Routing Operation

It is important to note that, although nodes have to switch between receiving and sending channels, which is the worst case scenario, node channel switching does not have to be synchronized for efficient performance. Rather, the distributed nature of the protocol operation allows for much flexibility, which ensures the availability of potential forwarding nodes without jeopardizing the downstream flow in any way. Moreover, it should be remembered that the protocol minimizes the multiple channel switching at the route selection phase, as discussed earlier in Section 3.4.

**Figure 7.**
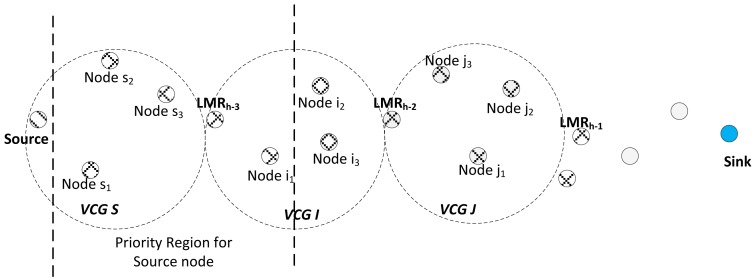
Illustration of the routing operation.

In order to elucidate this fact, we assume a source node, Sr, has a data packet to send, as illustrated in Figure 7; it will switch to channel Cs. Note that a node will only switch to its channel Cs when it has a packet to send, which means that all downstream nodes of the source's virtual cluster and that of the next virtual clusters will be automatically tuned to Cr0, as has been discussed in Section 3.5. It should also be remembered that, according to the node configuration as already discussed, Cs and Cr1 for VCG S and VCG I will be the same. A node will only switch to Cr1 if it had earlier received a data packet and subsequently returned a zero for its *I_DIFD_* operation for that packet. Otherwise, it will remain tuned to Cr0. This will become clearer in the second hop operation. Therefore, the source node broadcasts this packet on Cs. Assume all the nodes, *s*_1_, *s*_2_, *s*_3_, *i*_1_, *i*_2_ and *i*_3_, received the packet. However, only Nodes *s*_1_, *s*_2_, *s*_3_ and *i*_1_ return a one for their respective *I_DIFD_* operation, while Nodes *i*_2_ and *i*_3_ return a zero for the same operation. Thus, Nodes *i*_2_ and *i*_3_ will switch to Cr1.

**Figure 8.**
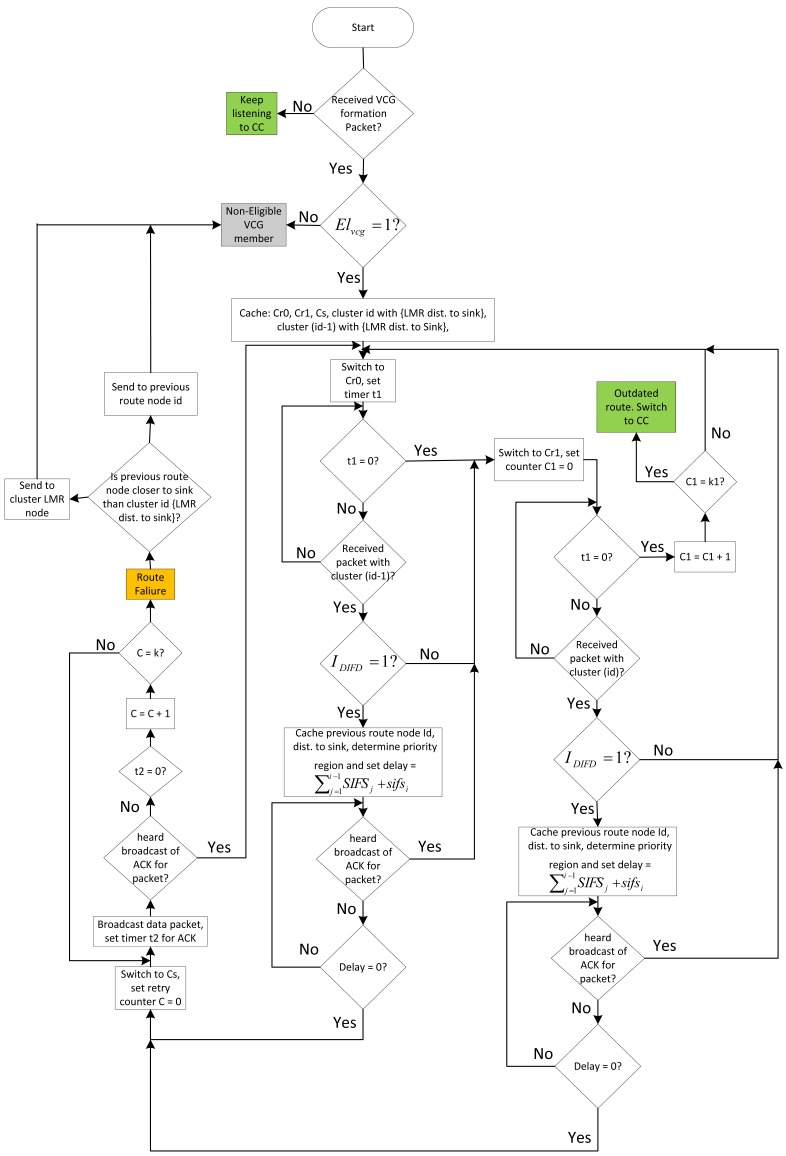
Flow chart for ROR node forwarding algorithm. CC, control channel; SIFS, short inter-frame space; DIFD, data initiative forwarding determination.

Then, Nodes *s*_1_, *s*_2_, *s*_3_ and *i*_1_ will perform the receiver contention prioritization operation. By virtue of Node *i*_1_ being closer to the sink than Nodes *s*_1_, *s*_2_, and *s*_3_, Node *i*_1_ wins this contention and sends its ACK packet first to the sink via Cs. After sending the acknowledgment, *i*_1_ then switches to Cs and broadcasts the packet. Remember that Nodes *i*_2_ and *i*_3_ are already tuned to their clusters, Cr1, while Nodes *j*_1_, *j*_2_ and *j*_3_ are all tuned to their clusters, Cr0, because they have not previously returned a zero for *I_DIFD_*. Already, since the channels, Cs, Cr1 for VCG I and Cr0 for VCG J, are all the same, all the nodes, *i*_2_, *i*_3_, *j*_1_, *j*_2_ and *j*_3_, will all take part in contending to forward the packet if their *I_DIFD_* operation returned a one. Notice that if a node that is already in its Cr1 channel subsequently returns a zero for *I_DIFD_*, it will automatically reverse back to Cr0. Thus, in this manner, the packet is forwarded effortlessly towards the sink. The full operation of the protocol is as depicted in the flow chart in Figure 8.

## 4. Operation Analysis

At this point, we have to mention that we do not seek to make a new analysis for geographical forwarding systems that utilize the joint SNR and distance metric [18]. Rather, we want to elucidate the behavior of the major components of ROR.

In line with this, for all channels in the chosen route that fulfill Equation (8), as specified from the application layer, the expected hop count to the sink can be approximated as [21] for the geographical forwarding operation of the protocol,

(13) $$E\left[n_{\text{hop}}\left(D\right)\right]≃\frac{D-d_{r}}{E\left[\varsigma \left(D\right)\right]}+1$$

where *D* is the distance from the source node to the sink node, *d_r_* represents the approximated transmission range and is the expected hop distance.

**Figure 9.**
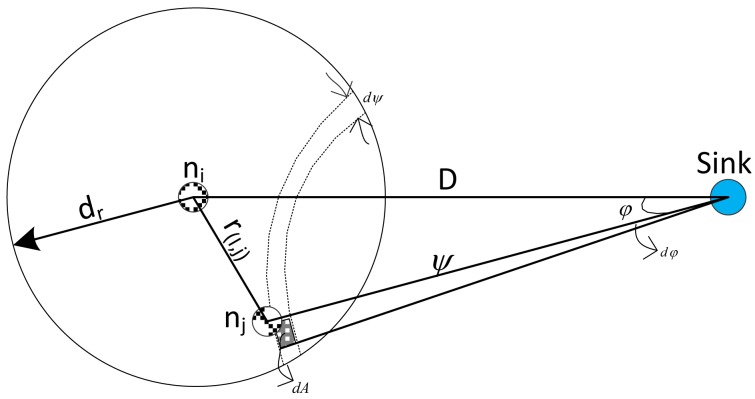
Reference model used for derivations.

As shown in Figure 9, we consider a node, *n_j_*, in the infinitesimal area *dA* = *dφdψ* with respect to the sink. The distance between *n_i_* and *n_j_*, *r*_(_*_i,j_*_)_ can be computed thusly:

(14) $$r_{\left(i,j\right)}=r(D,\psi ,\varphi )=\sqrt{\psi^{2}+D^{2}-2\psi D\cos\varphi }$$

Additionally, the expected hop distance, *E* [*ς*(*D*)], can also be computed as:

(15) $$E\left[\varsigma \left(D\right)\right]=\int_{\psi_{\min}}^{D}\int_{-\varphi_{\psi }}^{\varphi_{\psi }}r_{\left(r,j\right)}dP\{{\mathbb{N}}_{i}=j\}$$

where *dP* {ℕ*_i_* = *j*} is the probability that *n_j_* is selected at the next hop ℕ*_i_*, *ψ_min_* = *D* − *d_r_* and $\varphi_{\psi }=\text{acos}[(\psi^{2}+D^{2}-d_{r}^{2})/2\psi D]$. Remember that, according to Equation (12), this means that is the closest node to the sink among all nodes that fulfil $d_{sk}\leq d_{sk}^{fd}$ in the priority region that have retuned a one for the *I_DIFD_* operation.

Also, according to Equation (12)*n_j_* must satisfy *ξ_j_* ≥ *ξ_th_* to be selected as the next hop, which means that all nodes, *n_x_*, closer to the sink than *n_j_* received the packet with an SNR value less than the threshold, *i*.*e*., *ξ_x_* < *ξ_th_*. It is thus derived that the probability of selecting *n_j_* as the next hop is:

(16) $$dP\left\{{\mathbb{N}}_{i}=j\right\}=P\{N_{A(d\psi )}=1\}P\{\xi_{j}\geq \xi_{th}\}P\{r_{\left(j,s\right)}\geq \psi \}$$

*P*{*N_A_*_(_*_dψ_*_)_ = 1} represents the probability that there is a node inside the area, *A*(*dψ*), where *N_A_*_(_*_dψ_*_)_ is the number of nodes in the area, *dA*, at distance *ψ* from the sink. As *dψ* → 0, it can be approximated by:

(17) $$P\{N_{A(d\psi )}=1\}≃1-e^{-\rho \psi d\psi d\varphi }≃\rho \psi d\psi d\varphi$$

Since (*ρψdψdφ*) → 0 as *dψ* → 0 and *dφ* → 0, the approximation *e*^−^*^x^* ≃ 1 − *x* is used for the last hop.

On the other hand, *P*{*ξ_j_* ≥ *ξ_th_*} in Equation (17) is the probability that the received SNR of *n_j_* is above *ξ_th_*; while *P*{*r*_(_*_j,s_*_)_) ≥ *ψ*} is the probability that is at least a distance, *ψ*, from the sink, *s*. *P* {*ξ_j_* ≥ *ξ_th_*} and *P*{*r*_(_*_j,s_*_)_ ≥ *ψ*} can be derived with the log-normal channel model [11], which defines the power of a receiver at a distance, *r*, from a transmitter as:

(18) $$P_{r}\left(r\right)=P_{t}-PL(r_{0})-10\eta \log_{10}\left(\frac{r}{r_{0}}\right)+X_{\sigma }$$

where *P_t_* in dBm represents the transmit power, *PL* (*r*_0_) in dB is the path loss at a reference distance, *r*_0_, *η* represents the path loss exponent, while *X_σ_* represents the shadow fading component, with *X_σ_*∼ 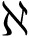 (0, *σ*). At the receiver, the signal-to-noise ratio (SNR) in dB is given by *ξ*(*r*) = *P_r_*(*r*) − *P_n_*. where *P_n_* is the noise power in dBm. Thus, taking into consideration the shadow fading component, *X_σ_*, *P*{*ξ_j_* ≥ *ξ_th_*} becomes:

(19) $$\begin{array}{ll} P\{\xi_{j}\geq \xi_{th}\} & =P\{X_{\sigma }\geq \beta (r_{\left(i,j\right)},\xi_{th})\}\\ & =Q\left(\frac{\beta (r_{\left(i,j\right)},\xi_{th})}{\sigma }\right) \end{array}$$

(20) $$\text{with}\beta \left(r_{\left(i,j\right)},\xi_{th}\right)=\xi_{th}+P_{n}-PL(r_{0})+10\eta \log_{10}\left(\frac{r_{\left(r,j\right)}}{r_{0}}\right)$$

and $Q(x)=\frac{1}{\sqrt{2\pi }(\int_{x}^{\infty }e^{-(t^{2}-t)})}dt$. Then, by representing the area wherein the nodes that are closer to the sink node than as *A*(*ψ*), *P*{*r*_(_*_j,s_*_)_ ≥ *ψ*} can subsequently be derived as:

(21) $$\begin{array}{ll} P\{r_{\left(j,s\right)}\geq \psi \} & =\overset{\infty }{\underset{i=0}{\text{∑}}}P\{N_{A(\psi )}=i\}p_{x}{\left(\psi \right)}^{i}\\ & =\overset{\infty }{\underset{i=0}{\text{∑}}}\frac{e^{-M(\psi )}M{\left(\psi \right)}^{i}}{i!}p_{x}{\left(\psi \right)}^{i}\\ & =e^{-M(\psi )(1-px(\psi ))} \end{array}$$

in this respect, *A*(*ψ*) represents the area of intersection of two circles, which cuts across two neighboring virtual clusters, with centers separated by *D* and with radii *d_r_* and *ψ*, respectively. Thus, *N_A_*_(_*_ψ_*_)_ represents the number of nodes in the area, *A*(*ψ*), while *M*(*ψ*) represents the average number of nodes in the area, which is = *ρA*(*ψ*). *p_x_*(*ψ*), which represents the probability that the received SNR, *ξ_x_*, for *n_x_* in *A*(*ψ*), is less than the SNR threshold, *ξ_th_*.

(22) $$p_{x}(\psi )=\rho \int_{\psi_{\min}}^{D}\int_{-\varphi_{\psi }}^{\varphi_{\psi }}\left[1-Q\left(\frac{\beta }{\sigma }\right)\right]\frac{1}{A(\psi )}d\varphi d\psi$$

where *ψ*_min_ = *D* − *d_r_*. Hence, the expected hop distance, *E* [*ς*(*D*)], can be derived by using Equations (16), (17), (19), (21) and (22) in Equation (15) to give:

(23) $$E\left[\varsigma \left(D\right)\right]=\rho \int_{\psi_{\min}}^{D}\int_{-\varphi_{\psi }}^{\varphi_{\psi }}\psi r_{\left(i,j\right)}Q\left(\frac{\beta }{\sigma }\right)e^{-M(\psi )(1-p_{x}(\psi ))}d\varphi d\psi$$

**Figure 10.**
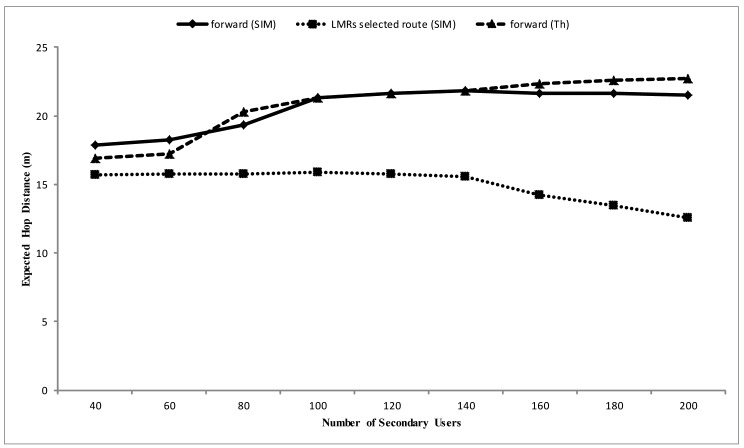
Expected hop distance *vs*. the number of CRSN nodes in the network.

Alongside this, a simulation set-up of a single stream flow from one source node to the sink allows a clearer view of the nature of the protocol. As observed in Figure 10, the expected distances between the LMR nodes along the selected routes gradually decrease as the number of nodes in the network increases. This is because the route request phase, as controlled by Equation (11), is basically a carefully controlled flooding operation to find all possible routes to the sink via CC. This strategy is typical to most previously proposed protocols, like SCEEM and ECR. With such schemes, it is not possible to stipulate the SNR reception limit criteria for packet forwarding. This is because both next hop and channel negotiation are already determined in the route request phase via CC, while the real packet forwarding process is done via a different data channel. However, via disconnecting the channel selection from the next hop selection in the route request and by the formation of VCGs along the path to source in the route select phase, the SNR reception limit criteria for packet forwarding can be used in order to maximize the opportunity of extending the next hop distance, since nodes in the transitional region usually exhibit the highest forwarding values for lossy links [18]. Thus, as observed from the Figure 10, the forwarding phase has the potential of selecting nodes that make more progress in the expected hop distance towards the sink, which, in summary, agrees with theoretical deduction. The value for *ξ_th_* used was 10 dB, while the values in Table 1 used for analytical purposes are gotten from Mica2 [40], which corresponds with our simulation environment, Prowler [34,41].

## 5. Performance Evaluation

Finally, we discuss the overall communication complexity of these solutions. We evaluate ROR and compare it with SCEEM in MATLAB based on the routing modeling application simulation environment (Rmase) [42,43], which is a plug-in for the probabilistic wireless network simulator (Prowler) [34,41], along with the cognitive radio and ROR routing layers we have developed. We present simulation results for a cognitive radio sensor topology of 40–200 nodes randomly deployed in a 100 × 100 *m*^2^ sensor field. The sink is located at the coordinates (80, 80). The relevant simulation parameters are given in Table 1. In each simulation, an event occurs in an event area located at coordinates (20, 20) with an event radius of 20 *m*^2^. Each source node reports its event information to the sink. At each node, seven (7) channels are available from which a selection is made during the RREQ phase, and all channels have the same bandwidth. None of the channels overlap, so that the packets transmitted on different channels do not interfere with each other. As highlighted in Section 3.1, both the PU and SU transmit power decays with distance based on the free-space path loss model, and the PU activity is modeled according to an ON and OFF Poisson state transition model [36,37], in which, both states represent the arrival or absence of the primary user, respectively. For the sake of this evaluation, performance is recorded for cases where *P_on_* is 0.2-0.8. In order to investigate the effect of the number of SUs in the presence of various intensities of PU activity and network load, each simulation is performed for the number of SUs values of 40:20:200. Each simulation lasts for 100 s, and the average of 10 trials for each of four different random topologies are shown along with their 80% confidence intervals.

In the evaluations, we investigate the following performance metrics:

- Throughput, which measures the time performance for any application, is the number of bits per second received at the sink. In calculating this metric, only unique packets are considered, since multiple copies of a packet can be received at the sink for certain protocols.
- Goodput measures the overall success or communication reliability of the network. It is the ratio between the total number of unique packets received at the sink and the total number of packets sent by all the source nodes.
- Latency is the time that passes between the time a packet is generated at a source node and the time it is received at the sink. This delay accounts for the queuing delay and the contention delay at the nodes, as well as specific protocol operation overhead.
- The loss rate measures the quality of the application. It is derived as the number of lost packets *vs*. the total expected number of packets for a destination. To obtain the loss rate, each packet is attached with a message identification, m, and a sequence identification, s. Assume that is the largest sequence identification of message, m, received at this node; when a new packet of m with s arrives, the loss for the packet is calculated by *s* − (*s′* + 1), if *s* > *s′*, otherwise. The total loss at a destination is then calculated by *L* = *s_i_l_i_*, where *l_i_* is the loss for the *i* − *th* packet. The loss rate at the sink is *L*/(*L* + *R*), where L is the number of packets lost, and R is the number of packets received.
- Energy efficiency is the ratio between the total number of packets received at the sink *vs*. the total energy consumption in the network.

### 5.1. ROR Evaluation

**Figure 11.**
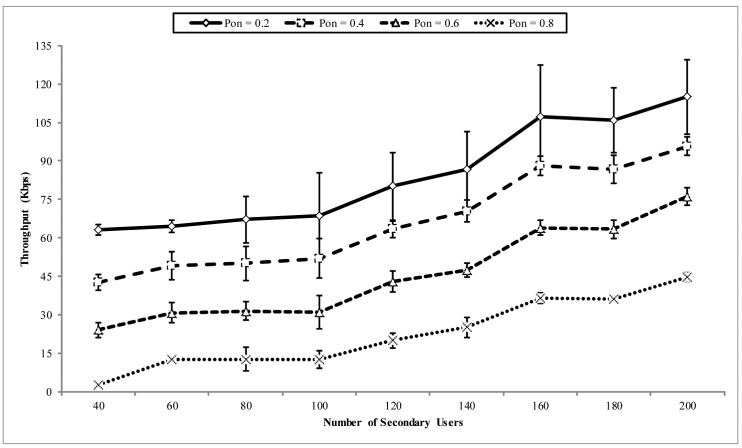
Throughput *vs*. number of CRSN nodes in the network.

In Figure 11, the throughput at the sink is shown. The x-axis shows the number of nodes (SUs) in the network, which also signifies the amount of loads generated in the network. This is because, as the network density increases, the number of source nodes in the event area that inject packets into the network also increases. The throughput performance is shown for different PU activity patterns. It can be observed that the throughput increases with the number of the nodes in the network. Likewise, as the frequency of the PU activity increases, a corresponding drop in the throughput is also experienced. This is because increased PU activity decreases the time SUs have to route packets, which subsequently affect the throughput at the sink. However, ROR is still able to efficiently ensure reasonable throughput at the sink in the presence of PU activity. This property is better observed in the goodput performance of ROR discussed below.

**Figure 12.**
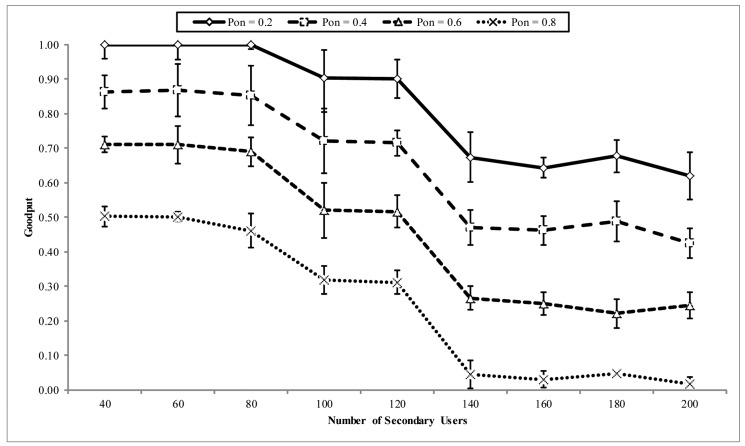
Goodput vs. number of CRSN nodes in the network.

**Figure 13.**
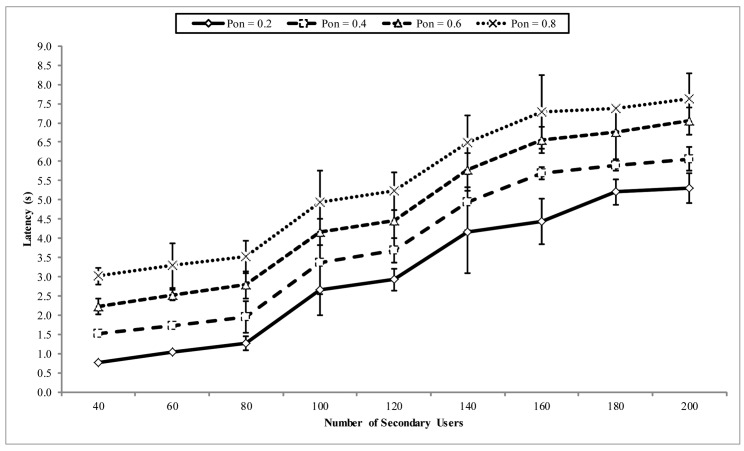
End-to-end latency vs. number of CRSN nodes in the network.

In Figure 12, the goodput performance is illustrated. This property is also shown at different PU activity patterns. In general, it can be observed that both PU activity and network load adversely affect the goodput performance, with PU activity having more of an effect than the network load. As the network load increases with the number of nodes in the network, eligible nodes in the priority region also increase. However, the effect of the implemented receiver contention prioritization scheme ensures the stability of the goodput as the load increases. Although the increase of nodes in the network is expected to increase the goodput, because of the increase of the opportunity for quality links, however, when more contending nodes compete in the priority regions, the probability of collision increases, which is responsible for the decline recorded at some points in the graph. Thus, it can be concluded that ROR ensures a stable reliability in the network of above 95% at moderate PU channel activity and network load. This reliability is greatly affected by PU activity and moderately affected by the network load. The performance in the throughput and goodput is exhibited in the end-to-end latency in the network. In Figure 13, the end-to-end latency is illustrated. Again, the end-to-end latency is shown for different PU activity patterns. The factors responsible for the gradual increase in the latency with network density are the increase in the number of eligible nodes participating in receiver contention and the PU activity. Thus, in ROR, latency increases with both network load and PU activity.

### 5.2. Comparative Evaluation

In order to compare ROR with other protocols, we have chosen to use SCEEM [23,24], because, until now, SCEEM is the only work that has properly addressed routing from the perspective of CRSN. Other works that have been cited in the review are not considered for comparison, because OSDRP [22] does not consider the unique constraints of CRSN, while SER [29] did not present a concise implementation of the MAC considered for the design. Similarly, ECR [28], which presents a similar clustering network model, does not mention how the clustering process is implemented. Thus, the best option will be to compare our approach with SCEEM [23,24].

**Figure 14.**
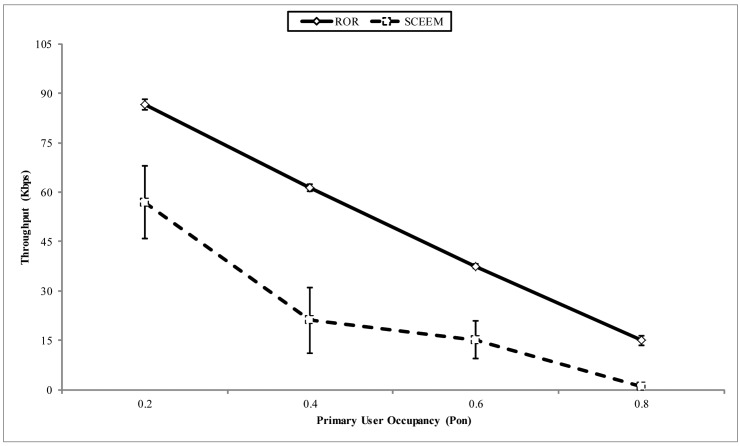
Throughput vs. primary user (PU) occupancy.

**Figure 15.**
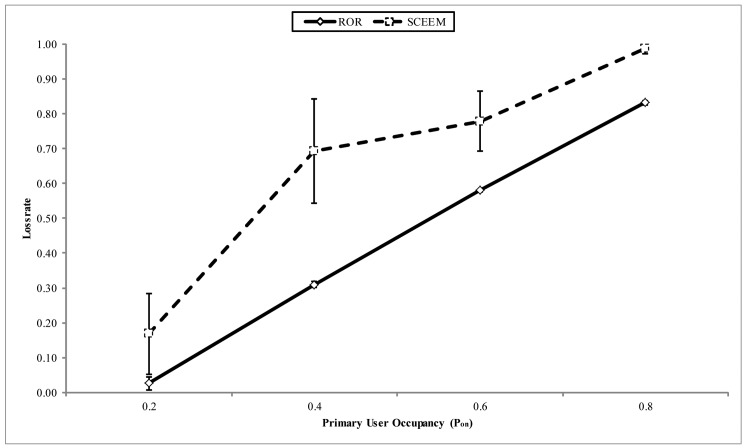
Loss rate vs. PU occupancy.

In Figure 14, the throughput at the sink is compared for both approaches. The x-axis shows the PU arrival pattern. It can be observed that the ROR strategy ensures that more packets arrive at the sink, as against SCEEM. The reason behind this performance for SCEEM can be explained thusly. In SCEEM, cluster heads aggregate packets to be sent to the sink from all cluster members. In turn, a path to the sink is negotiated with the cluster heads serving as intermediate routers. This strategy easily creates a bottleneck in the network, especially when the data meant for the sink is high. This attribute becomes multiplied with increased PU activity on the data channel. Thus, the protocol will experience more packet drops, as illustrated in Figure 15. This is contrary to decentralized routing strategy of ROR, which creates other paths among virtual cluster members to reduce packet dropping by avoiding the creation of potential hot spots in the network. Another important metric responsible for the performance of ROR is the SNR reliability metric integrated into the forwarding metric, as discussed below.

**Figure 16.**
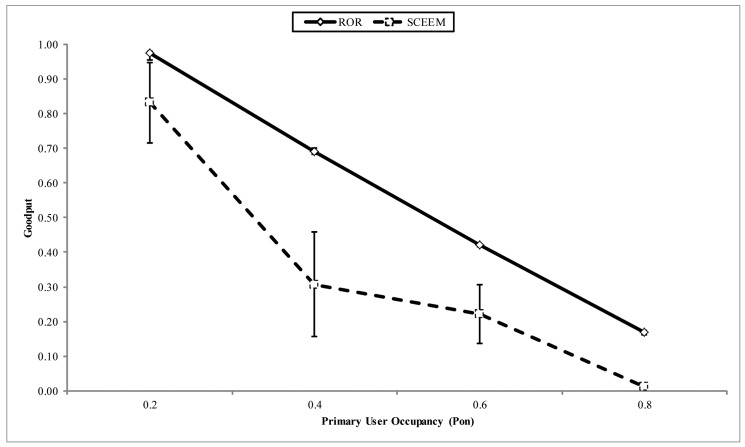
Goodput vs. PU occupancy.

In Figure 16, the goodput of both strategies is compared. It can be observed that an average of a 20% improvement is recorded by ROR in the reception rate when compared with SCEEM. Although, as we have already discussed above, the SNR reception metric is responsible for this performance by ROR, the prioritized receiver contention module also helps to reduce the contention and subsequently reduce collision during forwarding the packet towards the sink. On the other hand, although SCEEM also reduces the forwarding contention among nodes by clustering, the lack of instantaneous channel information often necessitates multiple re-tries in sending the packet to the next hop. This, in turn, has an adverse effect on the latency experienced by networks running SCEEM, as discussed next.

**Figure 17.**
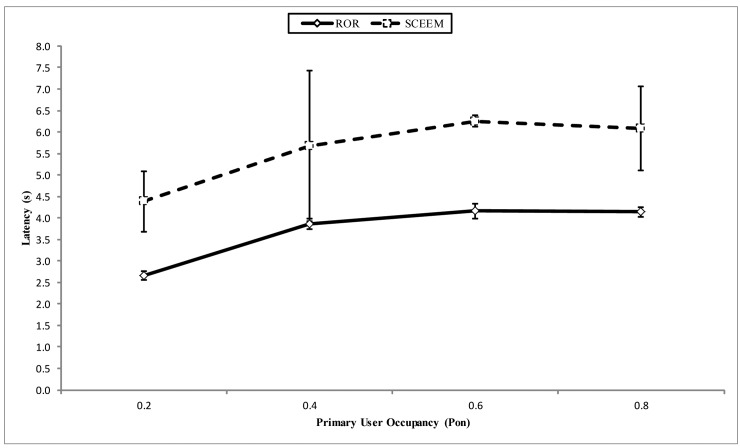
Latency vs. PU occupancy.

In Figure 17, the end-to-end latency of both approaches is compared. ROR outperforms SCEEM in this respect, basically because of the time taken to synchronize the SCEEM nodes in the TDMA communication-based clusters. Latency incurred in this process is fully part of the routing process and not part of the initialization of the network. The reason behind this is that the cluster head can switch to the data channel for packet forwarding within unused frame periods. Alongside this is the effect of maintaining a single path towards the sink to service accumulated packets from cluster members, which has an adverse effect on the queuing time of packets. On the other hand, ROR reduces latency via its geographical routing technique, which always seeks the best route that is closest to the sink, i.e., hop length extension. Moreover, there is no extra latency incurred for VCG formation, other than the normal initialization period for standard AODV. Furthermore, the queuing time is controlled by allowing other eligible nodes to route pending packets towards the sink.

**Figure 18.**
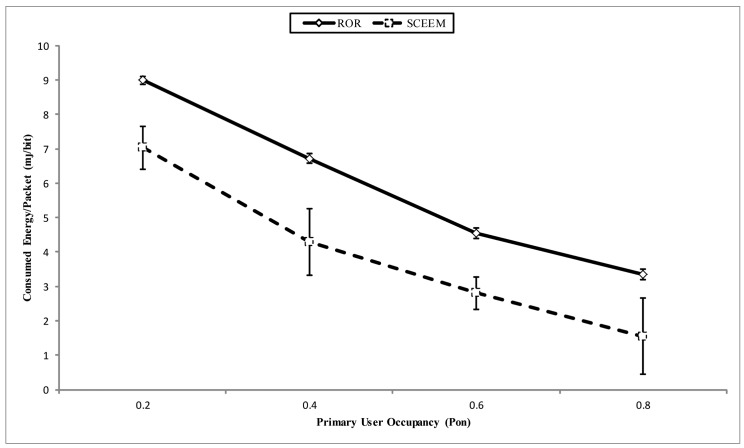
Consumed energy/packet vs. PU occupancy.

In Figure 18, the energy consumed per packet of both approaches is compared. Again, the energy consumed per packet for SCEEM exceeds that consumed in ROR. The factor responsible for this with respect to SCEEM can be traced to the cost of communication for synchronizing the nodes in the cluster and, subsequently, the intra-cluster communication. Alongside these, the cost incurred in idle listening when considered for the inter cluster communication makes the incurred energy cost exceed that of ROR. On the other hand, the hop length extension offered by ROR also decreases the energy consumption. Further, since ROR was designed to maximize the use of idle listening, the cost of idle listening is converted by the nodes to make critical network decisions without having to design separate algorithms to achieve same. Additionally, it should be noted that, unlike SCEEM in which cluster formation and maintenance are part of the routing process, the organization of the virtual clusters in ROR is fundamentally part of the network initialization stage, which is fully a part of the route request and reply stage. When compared to the standard AODV setup, the only difference is, again, the use of idle listening for nodes to decide if they are part of a virtual cluster or not. Thus, not only is ROR reliable, it is also an energy-efficient scheme that should be utilized for efficient routing in CRSNs.

## 6. Conclusions

In this paper, we have presented the ROR routing protocol, which addresses the issue of the reliability of node-to-node link quality in CRSN. Utilizing an energy-efficient technique, the strategy uses the common route reply strategy in traditional AODV to create virtual clusters around the next hop nodes of the selected routes. This further configures the nodes in the network in order to maximize the utilization of the geographical forwarding, while ensuring reliable node-to-node links at the point of data transfer. Theoretical analysis, which closely follows simulation results, shows that the strategy is able to transmit data to the sink in fewer hops than the closest node to the transmitting node metric, which is usually utilized for route search operations in similar schemes. This is because the strategy is designed to the select the nodes in the transitional region that exhibit the highest forwarding values. The simulation study shows that ROR can ensure a stable reliability in the network of above 95% at moderate PU channel activity and network load. This reliability is greatly affected by PU activity and moderately affected by the network load. Although the implemented receiver contention priority scheme can stabilize packet end-to-end latency at moderate network load and PU activity, ROR experiences a gradual increase in the latency with network density. This is primarily because of the increase in eligible nodes participating in receiver contention at each region and the PU activity. Further simulation studies for comparison have also revealed the favorable implications of adopting this strategy in terms of throughput, goodput, latency and energy. The scheme is also able to avoid the creation of routing hot spots in the network by its decentralized forwarding technique, thereby reducing packet drop due to network load, as against the compared approach. All these make ROR a favorable scheme that has the ability to improve the overall network quality of service for CRSNs.

## Figures and Tables

**Table 1.** Parameters.

*D*	*P_t_*	*PL*(*r*_0_)	*P_n_*	*σ*	*e*_(_*tx*)	*e*(*rx*)
100 *m*	0 *dBm*	55 *dB*	3	3.8	24 *mJ*	21 *mJ*
